# Study on mechanical and energy properties of rock-like specimens under the effect of loading rate

**DOI:** 10.1371/journal.pone.0349627

**Published:** 2026-07-01

**Authors:** Jingke Wu, Fuxing Xie

**Affiliations:** 1 School of Intelligent Manufacturing and Smart Transportation, Suzhou City University, Suzhou, China; 2 Beijing China Coal Mine Engineering Co., Ltd., Beijing, China; China University of Mining and Technology, CHINA

## Abstract

To investigate the effects of loading rate on the strength evolution, failure mechanism and energy properties of rock-like materials, rock-like specimens were prepared using similar materials, and tests under variable loading rate were conducted through uniaxial compression. Standard cylindrical specimens were fabricated with a fixed sand-binder ratio of 1:9 and three cement-gypsum ratios (1:9, 5:5 and 9:1), using river sand as aggregate and ordinary Portland cement and building gypsum as cementitious materials. The tests were conducted under two loading modes: displacement loading and force loading. The results show that the density of rock-like specimens increases linearly with the rise of cement-gypsum ratio; the stress-strain curves of specimens with different mix ratios all exhibit four stages, namely compaction, elasticity, yield and failure. The uniaxial compressive strength, elastic modulus and deformation modulus increase in the form of logarithmic function with the increase of loading rate, while the peak strain decreases logarithmically; all specimens present columnar splitting failure, and the stress concentration effect induced by force loading is more significant, resulting in a higher degree of failure compared with displacement loading. Moreover, the higher the loading rate, the more fully developed the fractures and the poorer the integrity of specimens. In terms of energy conversion, the lower the loading rate, the smaller the percentage of releasable elastic energy in the absorbed energy (the value of *U*_e_/*U* is greater than 70%), and the larger the percentage of dissipated energy in the absorbed energy (the value of *U*_d_/*U* is less than 30%). As the loading rate increases, *U*_e_/*U* increases as a logarithmic function, while *U*_d_/*U* exhibits an opposite trend. The findings of this study can provide a reference for support design and stability control in deep soft rock engineering.

## 1. Introduction

As a country rich in coal resources, China has witnessed the gradual depletion of shallow coal resources, pushing coal mining into deep areas. However, the high-stress and strongly disturbed geological environment of deep surrounding rock mass differs fundamentally from that of shallow strata. Soft rock roadways are prone to disasters such as large deformation and instability failure, which seriously threaten mining safety and engineering efficiency [1,2]. Therefore, an in-depth study on the mechanical response, failure mechanism and energy evolution law of soft rock under different loading conditions is of great practical significance for ensuring the stability of deep mining engineering.

Scholars at home and abroad have carried out extensive research on the mechanical properties of soft rock and the loading rate effect. In terms of the basic properties of soft rock, Kang et al. [3] systematically summarized the progress of control technologies for large deformation disasters of soft rock in China. Zhou [4], Xie [5], Li [6] et al. revealed the softening mechanism of soft rock under the water-rock interaction through microscopic tests. Zhou et al. [7,8] confirmed that the mechanical strength of soft rock decays exponentially with soaking time and tends to be stable. Liu et al. [9] established the constitutive model and Mohr-Coulomb failure criterion of fractured soft rock. In the research on the influence of loading rate. Chen Y. et al. [10] found that the tensile strength of coal-rock combinations is positively correlated with the loading rate. Li et al. [11] explored the influence of loading rate on the crack propagation and instability precursor of coal rock. Zhao et al. [12,13] pointed out that the smaller the loading rate, the more significant the brittle failure of blue sandstone. Bao et al. [14] found that the elastic modulus of granite at constant temperature shows a variation law of “decreasing first and then increasing” with the loading rate. Jin et al. [15] proposed that the plate-split surrounding rock is prone to overall instability at a low loading rate, while small block ejection occurs at a high loading rate.

However, there are many limitations in the field collection and processing of natural soft rock specimens, which are easy to lose their integrity due to disturbance and difficult to meet the requirements of test repeatability [16,17]. Rock-like specimens made of similar materials have the advantages of controllable composition, stable performance and convenient preparation, and their mechanical response is in good agreement with that of natural rock [18,19]. Luo et al. [20] studied the deformation mechanism of hard-soft interbedded rock-like materials based on the digital image correlation technology.

TRIMONOVA Mariia et al. investigated the rheological properties of rock-like materials under triaxial compression and uniaxial tension tests, and established an inelastic deformation model [21]. Zhang et al. analyzed the influence of different bedding dip angles on the mechanical properties and failure modes of shale, and proposed a reasonable method for determining mechanical parameters of deep soft rock [22]. Zhang et al. conducted triaxial compression tests on red-bed soft rock with various water contents, explored the effects of water content on its mechanical properties, failure modes, microstructures and damage mechanisms, and established a statistical damage constitutive model for water-bearing red-bed soft rock [23]. Si et al. developed a particle model for triaxial compression of composite rock with coplanar double fissures using numerical simulation software, and analyzed the failure characteristics and acoustic emission evolution rules of composite rock under different fissure dip angles, fissure lengths and confining pressures [24]. Li et al. pointed out that bedding planes and joints exert a significant influence on the characteristics of stress-strain curves and strength parameters. The presence of bedding planes inhibits the coalescence of cracks in the bridge area; as the joint dip angle increases, the influence of bedding planes on the failure characteristics of specimens decreases accordingly [25]. Fan et al. combined laboratory tests with numerical simulations of rock-like materials to analyze the distribution of principal stresses and principal strains around four-centered circular tunnels and horseshoe-shaped tunnels [26]. Lu et al. proposed that the geometric similarity of rock-like materials can be achieved by adjusting the water-binder ratio [27].

Most existing studies focus on single loading modes or rock-like materials with fixed mixing ratios. Research on the strength, failure and energy characteristics of rock-like specimens under different loading methods (displacement/force loading) and various cementing ratios remains insufficient. To address this gap, this study designs rock-like specimens with three cement-gypsum mixing ratios. Through uniaxial compression tests with variable loading rates, it systematically analyzes the effects of loading rate on the physical and mechanical parameters, failure modes and energy evolution laws of rock-like specimens, aiming to provide a scientific basis for disaster prevention and control in deep soft rock engineering.

## 2. Specimen preparation and experimental design

### 2.1. Material selection and mix proportion design

Natural river sand with particle sizes ranging from 0.0074 to 0.042 cm was used as aggregate. PO 42.5 ordinary Portland cement and building gypsum served as cementitious materials, with borax (0.3% by mass of total cementitious materials) as a retarder. The sand-binder ratio was fixed at 1:9. Three mix proportions were designed by adjusting the cement-gypsum ratio, labeled as mix 119 (1:9), mix 155 (5:5) and mix 191 (9:1). Eighteen specimens were prepared for each mix to ensure reliable parallel tests.

### 2.2. Specimen fabrication process

Standard cylindrical molds with a height of 100 mm and a diameter of 50 mm were used for specimen preparation. The process is outlined as follows:

Batching and mixing: Weigh materials per mix design, add an appropriate amount of distilled water, and stir thoroughly to eliminate agglomerates.

Batching and mixing: Materials were weighed in accordance with the mix proportion. An appropriate amount of distilled water was added, and the mixture was stirred thoroughly to eliminate agglomerates.

Molding and compaction: Pour the well-mixed slurry into molds, place molds on a vibrating table, and vibrate for 2 minutes to remove air bubbles and ensure uniform compaction.

Curing and demolding: Cure at room temperature for 24 hours, then carefully demold to avoid early-age cracking caused by disturbance.

Standard curing: Place demolded specimens in a constant-temperature and constant-humidity chamber for 7 days to ensure sufficient hydration of cementitious materials.

**Fig 1:**
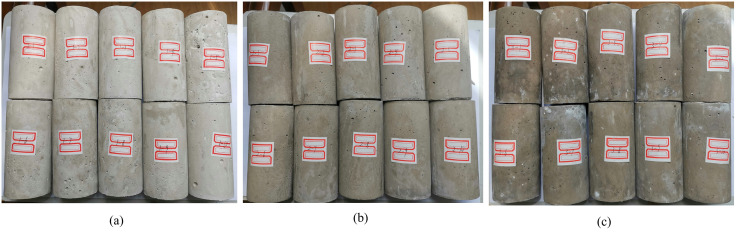
Rock-like specimens with different mix proportions (a) mix code 119, (b) mix code 155, (c) mix code 191.

Precision trimming: Grind both ends flat using a grinding machine to ensure end flatness error is less than 0.02 mm, meeting dimensional accuracy requirements for uniaxial compression tests (Fig 1).

### 2.3. Experimental setup and scheme design

#### 2.3.1. Experimental setup.

**Fig 2:**
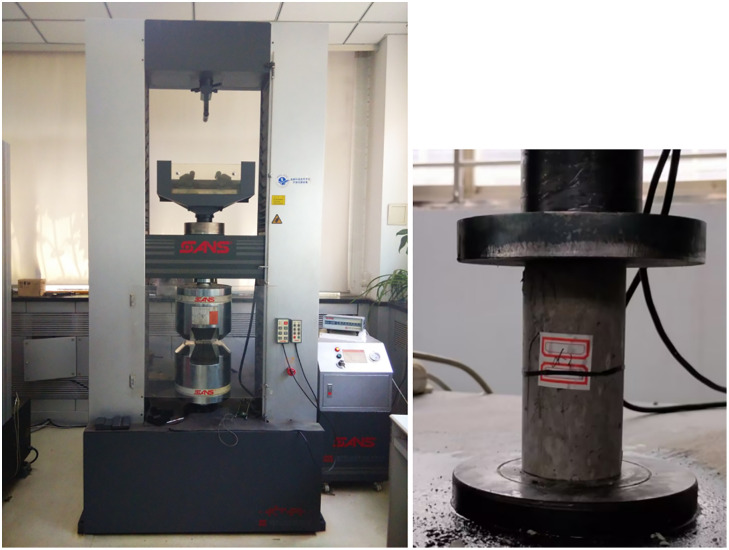
CMT5605 large-frame microcomputer-controlled electronic universal testing machine.

Uniaxial compression tests were carried out using a CMT5605 large-frame microcomputer-controlled electronic universal testing machine (Fig 2). The maximum loading force of the testing machine is 600 kN, with a displacement measurement accuracy of ±0.01 mm and a force measurement accuracy of ±0.5% FS. It supports both displacement-controlled and force-controlled loading modes, and can collect data such as pressure, displacement and time in real time. The sampling frequency was set to 10 Hz to ensure data continuity and accuracy.

#### 2.3.2. Loading scheme.

To compare the effects of different loading rates, the loading scheme was designed as follows:

Displacement loading (mix code 119, 155): The loading rates were set at 0.05, 0.1, 0.2, 0.5 and 1.0 mm/min, covering the loading range from low to medium-high rates to simulate different construction disturbance rates in engineering practice.

Force loading (mix code 191): The loading rates were set at 0.02, 0.05, 0.1, 0.2 and 0.5 kN/s, which forms a corresponding mechanical response relationship with the displacement loading rate range.

## 3. Mechanical and deformation characteristics of rock-like specimens under variable loading rates

### 3.1. Physical properties of specimens

**Fig 3:**
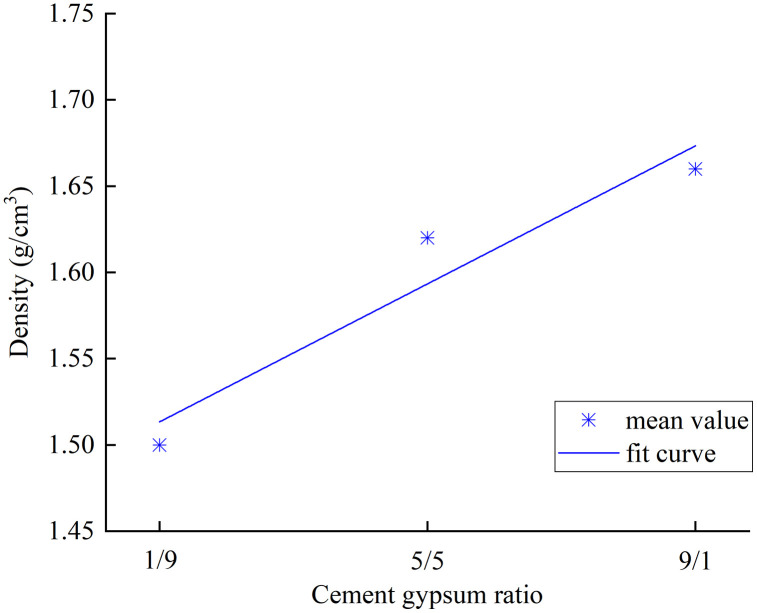
Variation curve of density with cement-to-gypsum ratio.

The diameter and height of the specimens were measured with a vernier caliper, and the mass was measured with an electronic balance, from which the density of the specimens was calculated, as shown in Table 1. The results demonstrate that the density of rock-like specimens increases linearly with rising cement content and falling gypsum content: the average density for mix code 119 is 1.50 g/cm³, 1.62 g/cm³for mix code 155, and 1.66 g/cm³for mix code 191. This was because the density of cement was significantly higher than that of gypsum. According to the superposition principle of mixed material density, the increase in the proportion of cement led to an increase in the mass of specimens per unit volume. Moreover, the density growth trend showed a good linear correlation with the cement-gypsum ratio (*R*² = 0.987), which verified the rationality of the mix ratio design, as shown in Fig 3.

**Table 1:** Density of specimen.

Mix code	Density range (g/cm^3^)	Average density (g/cm^3^)
119	1.48 ~ 1.58	1.50
155	1.56 ~ 1.73	1.62
191	1.57 ~ 1.74	1.66

### 3.2. Characteristics of Stress-Strain Curves

**Fig 4:**
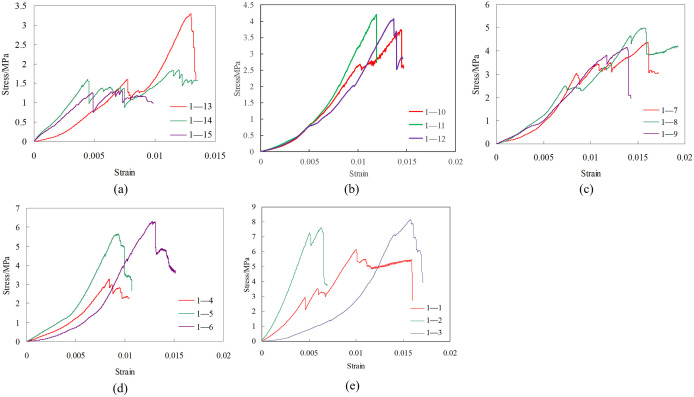
Stress-strain curves of rock-like specimens with mix code 119 under different loading rates (a) 0.05 mm/min, (b) 0.1 mm/min, (c) 0.2 mm/min, (d) 0.5 mm/min, (e) 1.0 mm/min.

The complete stress-strain curves of rock-like specimens with Mix No. 119 under variable loading rates are presented in Fig 4.

As shown in Fig 4, the peak strength of specimen with mix number 119 exhibits a significant decreasing trend with the reduction of loading rate. At a loading rate of 1.0 mm/min, the peak strength ranges from 6.15 to 8.16 MPa with an average value of 7.31 MPa. When the loading rate decreases to 0.5, 0.2, 0.1 and 0.05 mm/min, the average peak strengths are 5.17, 5.07, 4.01 and 2.15 MPa, respectively, representing decreases of 29.27%, 30.64%, 45.14% and 70.59% compared with that at 1.0 mm/min. At low loading rates, internal microcracks and pores in the specimen have sufficient time to propagate and coalesce, leading to progressive damage accumulation, which significantly weakens the overall bearing capacity of the material and results in a substantial reduction in strength.

**Fig 5:**
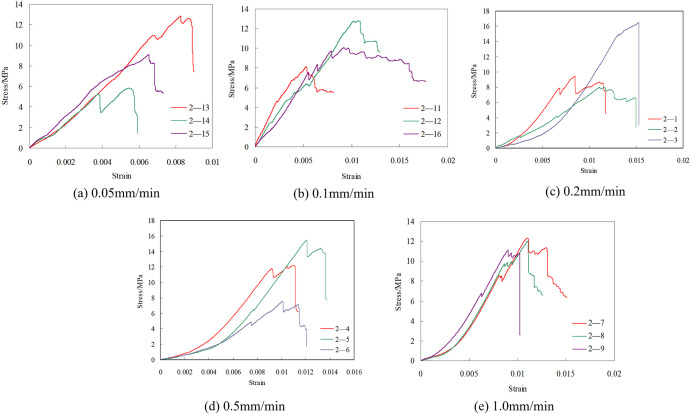
Stress-strain curves of rock-like specimens with mix code 155 under different loading rates (a) 0.05 mm/min, (b) 0.1 mm/min, (c) 0.2 mm/min, (d) 0.5 mm/min, (e) 1.0 mm/min.

The complete stress-strain curves of rock-like specimens with mix number 155 under variable loading rates are illustrated in Fig 5.

It can be seen from Fig 5 that the peak strength of the specimens with Mix No. 155 also decreases with the reduction of loading rate, yet the attenuation amplitude is smaller than that of the specimens with mix code 119. At a loading rate of 1.0 mm/min, the peak strength ranges from 11.12 to 12.36 MPa (with an average value of 11.83 MPa). At loading rates of 0.5, 0.2, 0.1 and 0.05 mm/min, the average peak strengths are 11.74, 11.32, 10.33 and 9.26 MPa respectively, which are 0.76%, 4.31%, 12.67% and 21.72% lower than that at 1.0 mm/min. This phenomenon stems from the balanced content of cement and gypsum in this mix ratio, which results in a more stable cementing system. At low loading rates, in addition to damage accumulation caused by the propagation of microcracks, changes in strain rate sensitivity and energy distribution patterns further intensify the strength degradation, though the overall impact is relatively weak.

**Fig 6:**
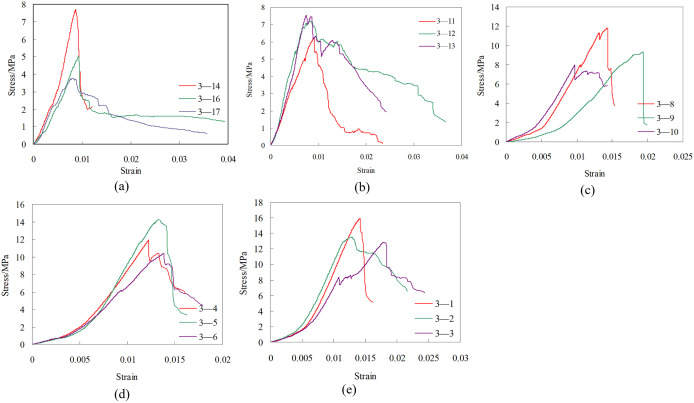
Stress-strain curves of rock-like specimens with mix code 191 under different loading rates(a) 0.02kN/s, (b) 0.05kN/s, (c) 0.1kN/s, (d) 0.2kN/s, (e) 0.5kN/s.

The complete stress-strain curves of rock-like specimens with mix No. 191 under variable loading rates are shown in Fig 6.

As shown in Fig 6, the peak strength of specimen with mix number 191 under force loading mode exhibits an obvious decreasing trend with the reduction of loading rate. At a loading rate of 0.5 kN/s, the peak strength ranges from 12.88 to 15.95 MPa with an average value of 14.11 MPa. When the loading rate decreases to 0.2, 0.1, 0.05 and 0.02 kN/s, the average peak strengths are 12.19, 9.72, 6.97 and 5.32 MPa, respectively, representing decreases of 13.61%, 31.11%, 50.60% and 62.30% compared with that at 0.5 kN/s. The stress concentration effect induced by force loading leads to more severe damage evolution inside the specimen. At low loading rates, the full coalescence of microcracks, combined with the effects of strain rate sensitivity and energy distribution, results in a substantially greater strength degradation than that observed in the specimen with mix number 155.

**Fig 7:**
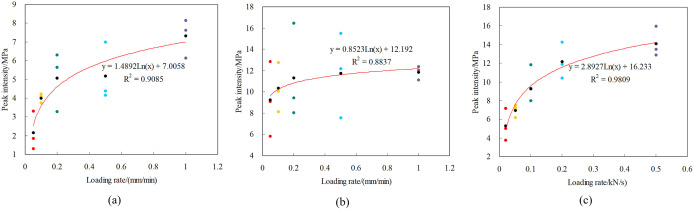
Relationship curves between peak strength and loading rate for specimens with different mix proportions (a) mix code 119, (b) mix code 155, (c) mix code 191.

**Fig 8:**
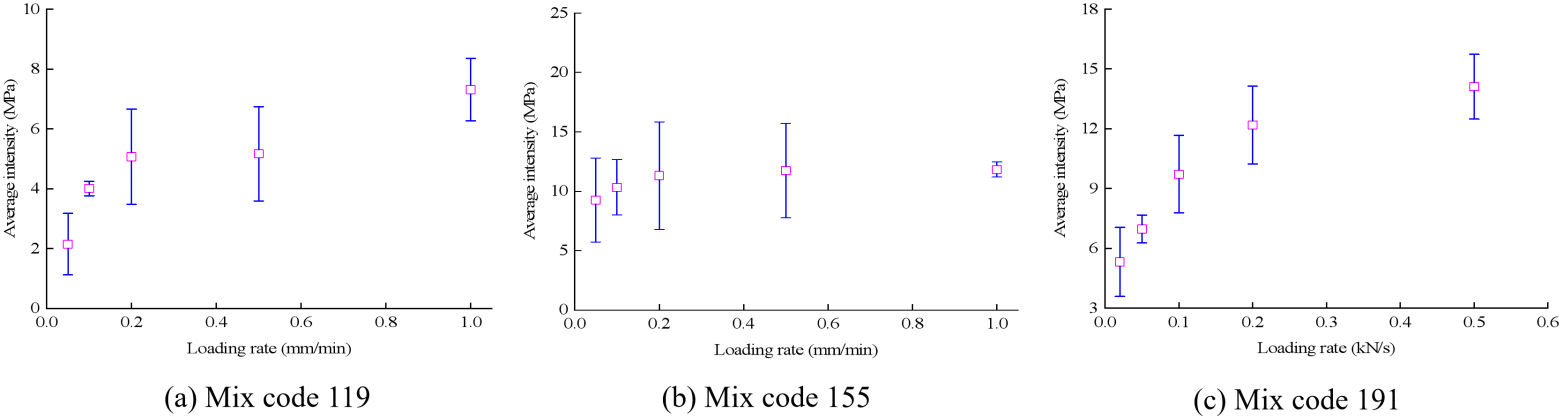
Standard error bars of peak intensity modulus for specimens with various mix proportions (a) mix code 119, (b) mix code 155, (c) mix code 191.

Combining the results from Fig 4, Fig 5, and Fig 6, it can be seen that the stress-strain curves of rock-like specimens with different mix ratios all exhibit typical four-stage characteristics: the compaction stage, where the primary micropores inside the specimens are compacted and closed, and the stress-strain curve presents a concave-up shape; the elastic stage, where stress and strain show an approximately linear relationship with a stable curve slope (corresponding to the elastic modulus); the yield stage, where microcracks initiate and propagate, deformation transitions from elasticity to plasticity, and the curve shows a concave-down shape; the failure stage, where the stress drops rapidly after reaching the peak value, the specimen loses stability and fails, and then exhibits residual bearing capacity. Fitting analysis of the peak strength and loading rate (Fig 7 and Fig 8) reveals that they satisfy a logarithmic function relationship (the fitting degree *R*^2^ is all greater than 0.92), i.e., the peak strength of the specimens increases logarithmically with the increase of loading rate, which is consistent with the rate sensitivity characteristic of rock-like materials.

### 3.3. Evolution patterns of deformation parameters

#### 3.2.1. Elastic modulus.

**Fig 9:**
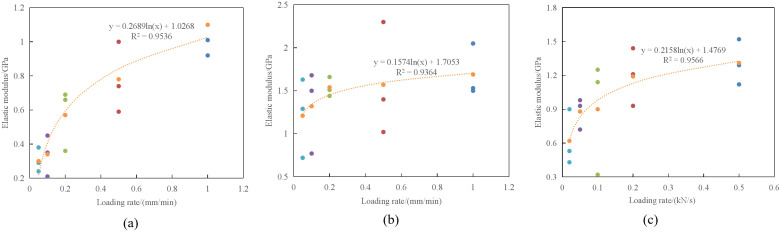
Variation curves of elastic modulus with loading rate (a) mix code 119, (b) mix code 155, (c) mix code 191.

Fig 9 shows the relationship curves between the elastic modulus of the rock-like specimens and the loading rate under different mix proportions.

**Fig 10:**
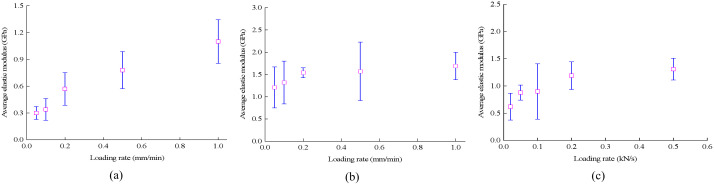
Standard error bars of elastic modulus for specimens with various mix proportions (a) mix code 119, (b) mix code 155, (c) mix code 191.

As a key parameter characterizing the ability of a material to resist elastic deformation, the variation law of elastic modulus with loading rate is shown in Fig 9 and Fig 10. The results indicate that the elastic modulus increases logarithmically with the increase of loading rate, with all coefficients of determination (*R*²) greater than 0.90. For mix number 119: the elastic modulus is 0.30 GPa at a loading rate of 0.05 mm/min and increases to 1.10 GPa at 1.0 mm/min, corresponding to an increase of 267%; the elastic deformation capacity of specimens with low cement content is more rate-sensitive. For mix number 155: the elastic modulus is 1.21 GPa at 0.05 mm/min and rises to 1.69 GPa at 1.0 mm/min, with an increase of 39.7%; the balanced mix ratio endows the material with more stable elastic properties. For mix number 191: the elastic modulus is 0.62 GPa at 0.02 kN/s and increases to 1.31 GPa at 0.5 kN/s, an increase of 111.3%, and the growth rate of elastic modulus under force loading mode is higher than that under displacement loading mode.

It can be concluded from the above analysis that at high loading rates, the arrangement of atoms and molecules inside the material becomes more compact, enhancing the elastic deformation capacity and resulting in an increase in elastic modulus. At low loading rates, plastic deformation develops sufficiently, leading to a relatively low elastic modulus. This phenomenon is consistent with the rate-dependent theory of material mechanics.

#### 3.3.2. Deformation Modulus.

**Fig 11:**
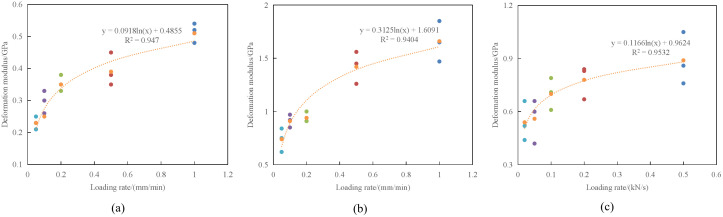
Deformation modulus-loading rate curves (a) mix code 119, (b) mix code 155, (c) mix code 191.

Fig 11 shows the relationship curves between the deformation modulus (*E*_50_) of the rock-like specimens and the loading rate under different mix proportions. The deformation modulus *E*_50_ is defined as the slope of the line connecting the origin to the point corresponding to 50% of the peak stress on the stress-strain curve.

**Fig 12:**
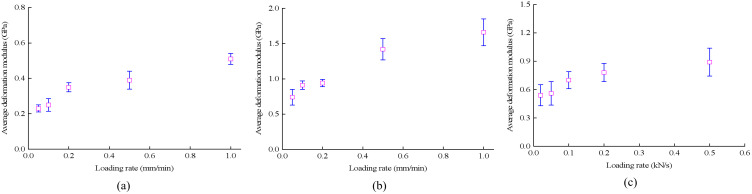
Standard error bars of deformation modulus for specimens with various mix proportions (a) mix code 119, (b) mix code 155, (c) mix code 191.

As a key parameter characterizing the ability of a material to resist elastic deformation, the variation of deformation modulus with loading rate is illustrated in Fig 11 and Fig 12. The results demonstrate that the deformation modulus increases logarithmically with the increase of loading rate, with all coefficients of determination (*R*²) greater than 0.90. For mix number 119: the deformation modulus is 0.23 GPa at a loading rate of 0.05 mm/min and rises to 0.51 GPa at 1.0 mm/min, corresponding to an increase of 122%; the deformation deformation capacity of specimens with low cement content is more rate-sensitive. For mix number 155: the deformation modulus is 0.74 GPa at 0.05 mm/min and increases to 1.66 GPa at 1.0 mm/min, with an increase of 124%; the balanced mix ratio imparts more stable elastic properties to the material. For mix number 191: the deformation modulus is 0.54 GPa at a loading rate of 0.02 kN/s and rises to 0.89 GPa at 0.5 kN/s, representing an increase of 64.81%, and the growth rate of deformation modulus under force loading mode is lower than that under displacement loading mode.

### 3.4. Analysis of specimen failure modes

**Fig 13:**
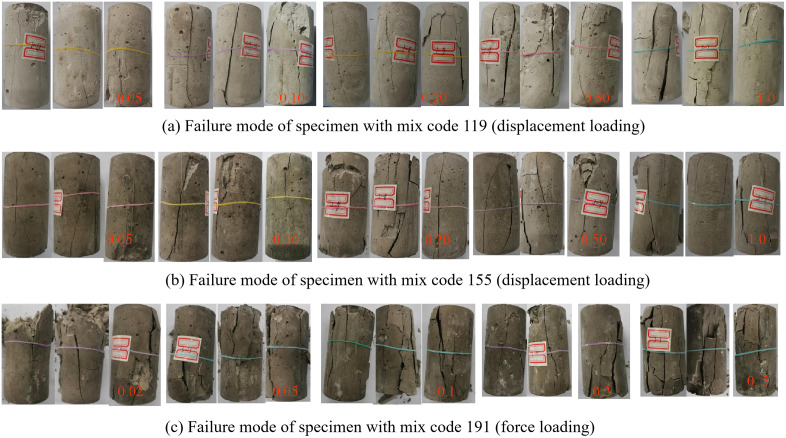
Failure modes of rock-like specimens under different mix proportions (a) mix code 119, (b) mix code 155, (c) mix code 191.

The failure modes of rock-like specimens with different mix numbers are shown in Fig 13.

All rock-like specimens exhibited columnar splitting failure under uniaxial compression, i.e., multiple macroscopic cracks parallel to the loading direction were generated along the axial direction, and the specimens were split into several columnar blocks along these cracks (Fig 13). This failure mode is consistent with the tensile failure mechanism of rocks under uniaxial compression (transverse tensile cracking induced by the Poisson effect). The failure mode is affected by the coupling of loading mode and loading rate, with the specific characteristics described as follows:

(1) Influence of loading mode

The degree of failure induced by force loading (mix number 191) was significantly greater than that by displacement loading (mix numbers 119 and 155). Specimens under displacement loading maintained good integrity after failure, with only 1 ~ 2 main cracks and weak crack coalescence. This is because displacement loading has a buffering effect, leading to gentle stress transfer and sufficient time for stress adjustment inside the specimens. In contrast, specimens under force loading suffered severe fragmentation; some specimen ends presented a powdery morphology, with a large number of main cracks and strong coalescence, and even block ejection occurred. This is due to the direct application of load on the specimens under force loading, resulting in a more pronounced stress concentration effect, which easily triggers instantaneous energy release and causes severe failure.

(2) Influence of loading rate

With the increase of loading rate, the crack development degree of specimens after failure increased significantly. At low loading rates (e.g., 0.05 mm/min, 0.02 kN/s), specimens remained relatively intact after failure, with only a small number of fine cracks and crack width is less than 0.5 mm. This is because microcracks had sufficient time to propagate and coalesce slowly at low rates, leading to uniform damage accumulation. At high loading rates (e.g., 1.0 mm/min, 0.5 kN/s), a large number of coalescent cracks were generated in the specimens, with crack widths up to 2 ~ 3 mm, and surface spalling occurred in some parts, resulting in extremely poor integrity. This is attributed to the fact that the crack propagation rate exceeded the energy dissipation rate at high rates, and the concentrated energy release led to rapid crack development. The evolution law of failure modes further verifies the regulatory effect of loading rate on the failure mechanism of rock-like specimens, providing a theoretical basis for reducing surrounding rock failure by controlling construction rates in engineering practice.

## 4. Analysis of energy characteristics

### 4.1. Theoretical basis

Based on the first law of thermodynamics, and considering with negligible heat exchange between the specimen and its surroundings and the environment during uniaxial compression, the total energy *U* input by the testing machine (i.e., (the energy absorbed by the specimen) the energy absorbed by the specimen) equals the sum of the releasable elastic energy *U*_e_ and the dissipated energy *U*_d_ [28]. That is:


$$\begin{array}{c} U=U_{\text{e}}+U_{\text{d}} \end{array}$$ (1)

Where, *U* is total energy (*M*J·m^-3^); *U*_e_ is releasable elastic energy (*M*J·m^-3^); *U*_d_ is dissipated energy (MJ·m^-3^).

The total energy *U* is calculated as the area enclosed by the stress-strain curve and the strain axis, determined using the trapezoidal integration method:


$$\begin{array}{c} U=\sum_{\text{i}=0}^{\text{n}}\int \frac{\text{1}}{\text{2}}(\overset{\text{i}}{\underset{\text{1}}{\sigma }}+\overset{\text{i}+1}{\underset{\text{1}}{\sigma }}\text{)(}\overset{\text{i}+1}{\underset{\text{1}}{\varepsilon }}-\overset{\text{i}}{\underset{\text{1}}{\varepsilon }}\text{)} \end{array}$$ (2)

Where, *n* is the number of axial stress-strain curve segments selected for calculation during any period of the experiment; i is sampling point; *σ* is the stress at the sampling point (MPa); ε is the strain at the sampling point.

The releasable elastic energy *U*_e_ is calculated based on the elastic modulus, assuming the material exhibits linear elastic characteristics before reaching the peak strength:


$$\begin{array}{c} U_{\text{e}}=\frac{\overset{\text{2}}{\underset{\text{1}}{\sigma }}}{2E_{\text{0}}} \end{array}$$ (3)

Where, *σ* is the stress at the sampling point (MPa); *E*_0_ is the elastic modulus (GPa).

The dissipated energy *U*_d_ is calculated as the difference between the total energy and the releasable elastic energy:


$$\begin{array}{c} U_{\text{d}}=U-U_{e} \end{array}$$ (4)

### 4.2. Energy evolution patterns

**Fig 14:**
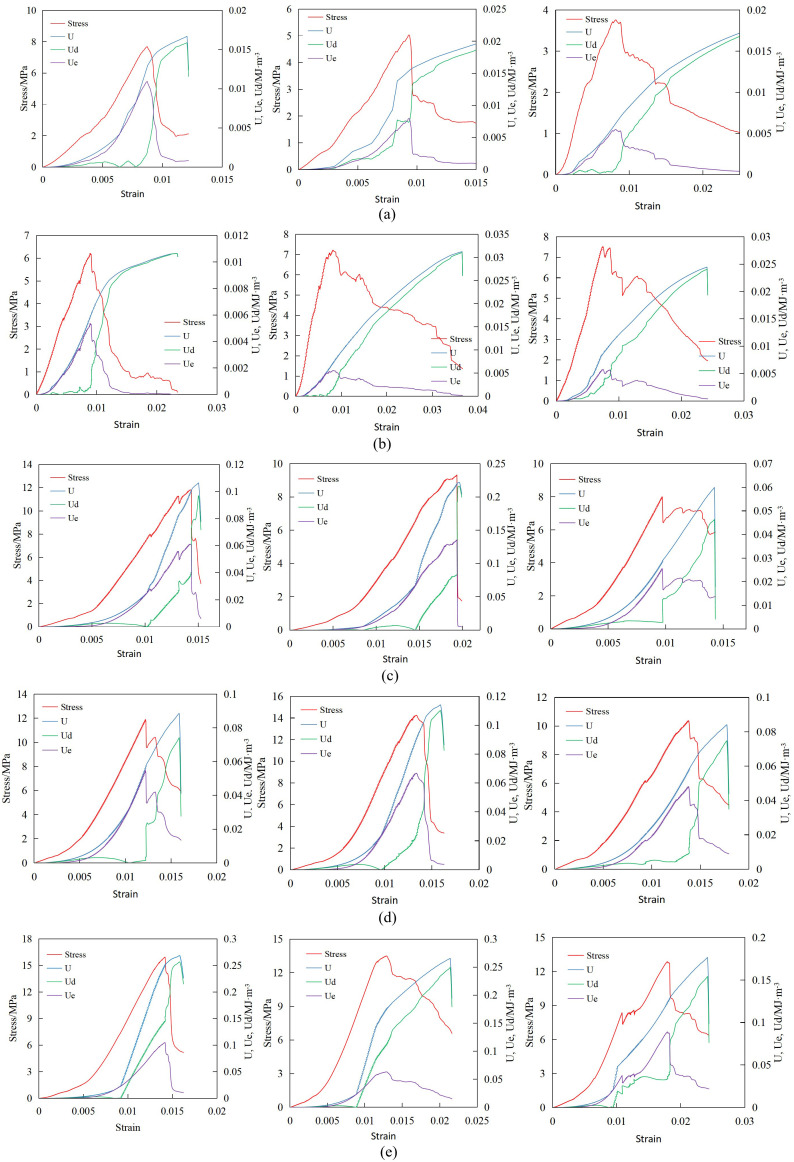
Stress-strain-energy relationship curves of the specimen under mix code 191(a) 0.02kN/s, (b) 0.05kN/s, (c) 0.1kN/s, (d) 0.2kN/s, (e) 0.5kN/s.

Taking mix code 191 as an example, the stress-strain-energy relationship curves of the specimens under different loading rates are shown in Fig 14. In the initial loading stage (compaction stage and early elastic stage), the total energy increased slowly and was primarily stored within the specimen in the form of elastic energy. The energy growth rate was consistent with the stress growth rate. From the late elastic stage to the yield stage, the growth rate of total energy accelerated, and the proportion of elastic energy gradually reached its peak. During this phase, microcracks began to initiate inside the specimen, with a small amount of energy being consumed for crack propagation (dissipated energy). After the peak strength, the elastic energy was rapidly released, and the dissipated energy increased significantly, corresponding to the intense failure process of the specimen. The total energy decreased with the stress drop, synchronizing with the mechanical response in the failure stage.

The energy parameters under different loading rates are shown in Table 2 (taking mix code 191 as an example). The results indicate that the lower the loading rate, the smaller the proportion of releasable elastic energy (*U*_e_/*U*) and the larger the proportion of dissipated energy (*U*_d_/*U*). At a loading rate of 0.02 kN/s, *U*_e_/*U* is 70.85% and *U*_d_/*U* is 29.15%; at a loading rate of 0.5 kN/s, *U*_e_/*U* is 82.46% and *U*_d_/*U* is 17.54%. *U*_e_/*U* increases logarithmically with increasing loading rate (*R*^2^ = 0.93), while *U*_d_/*U* decreases logarithmically with increasing loading rate (*R*^2^ = 0.91). This demonstrates that the loading rate influences the failure behavior of the specimens by regulating the energy distribution pattern.

**Table 2:** Energy parameters at peak stress for specimens under mix code 191.

*U*/MJ·m^-3^	*U*_e_/MJ·m^-3^	*U*_d_/MJ·m^-3^	*U*_e_/*U*(%)	*U*_d_/*U*(%)	loading rate/(kN/s)
0.2076	0.1426	0.0650	82.46	17.54	0.5
0.1037	0.0984	0.0053			
0.1021	0.0999	0.0022			
0.0567	0.0550	0.0017	81.49	18.51	0.2
0.0907	0.0664	0.0243			
0.0574	0.0455	0.0119			
0.0951	0.0759	0.0192	76.82	23.18	0.1
0.2102	0.1525	0.0577			
0.0295	0.0288	0.0007			
0.0060	0.0053	0.0007	76.61	23.39	0.05
0.0072	0.0056	0.0016			
0.0086	0.0058	0.0028			
0.0128	0.0109	0.0019	70.85	29.15	0.02
0.0153	0.0079	0.0074			
0.0062	0.0055	0.0007			

At low loading rates, microcracks inside the specimen propagate slowly, with more energy being consumed for crack propagation and plastic deformation, resulting in a higher proportion of dissipated energy. At high loading rates, crack propagation is insufficient, and energy is primarily stored in the form of elastic energy, which is released intensively after the peak strength, leading to a significant increase in *U*e/*U*. This trend is consistent with the evolution characteristics of the failure morphology, verifying the coupling relationship between energy transformation and the failure mechanism.

**Fig 15:**
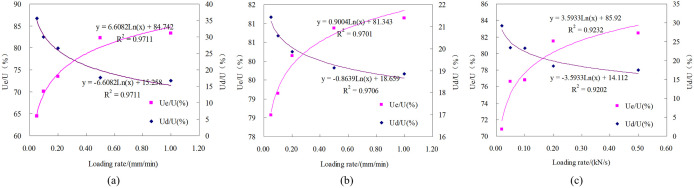
Relationship curves between Ue/U and Ud/U at peak stress and loading rate for rock-like specimens under different mix proportions (a) mix code 119, (b) mix code 155, (c) mix code 191.

Based on the results in Table 2, curves of *U*_e_/*U* and *U*_d_/*U* versus loading rate were plotted, as shown in Fig 15.

As shown in Fig 15, the lower the loading rate, the smaller the percentage of releasable elastic energy relative to the absorbed energy (the value of *U*_e_/*U* is greater than 70%), and the larger the percentage of dissipated energy relative to the absorbed energy (the value of *U*_d_/*U* is less than 30%). As the loading rate increases, *U*_e_/*U* increases logarithmically, while the trend for *U*_d_/*U* is the opposite.

## 5. Discussion

The uniaxial compression test was conducted to investigate the effects of loading rate on the mechanical properties, failure modes and energy evolution characteristics of rock-like specimens, providing fundamental experimental evidence for support design and stability control of deep soft rock engineering. However, there exists an essential difference between the uniaxial stress state and the actual triaxial compressive environment of deep soft rocks.

The linear increasing variation of the density of rock-like specimens with the cement-gypsum ratio provides a quantitative basis for preparing similar materials meeting specific density requirements. In the physical simulation of deep rock engineering, the density similarity between rock-like specimens and natural rocks can be achieved by adjusting the cement-gypsum ratio according to the density index of natural surrounding rocks, thereby improving the simulation accuracy and result reliability of model tests. The logarithmic growth of mechanical parameters of rock-like specimens with loading rate essentially reflects the rate-dependent characteristics of materials. At high loading rates, the propagation velocity of stress waves inside the specimens is faster than that of crack propagation, leading to the mechanical response of “increased rigidity and reduced plasticity” of the material; at low loading rates, microcracks have sufficient time to initiate, propagate and coalesce, making the plastic characteristics of the material more prominent. This result indicates that in deep high-stress environments, the construction rate of roadway excavation and support directly affects the stability of surrounding rocks. Rapid excavation easily induces brittle instability of surrounding rocks. Therefore, measures such as stepwise excavation and timely support should be adopted in engineering to reduce the hazards caused by strong disturbances. The coupling relationship between failure modes and energy evolution shows that both force loading mode and displacement loading mode can trigger concentrated energy release and accelerate the failure degree of specimens. In the support of on-site soft rock roadways, strong impact and high-disturbance construction should be avoided as much as possible. The energy release rate can be delayed by adding buffer structures, optimizing support stiffness and timing, etc., to mitigate the damage to surrounding rocks. Meanwhile, the law that the degree of crack development intensifies with the increase of loading rate can provide a reference for suppressing the damage and propagation of surrounding rocks by controlling the construction rate in engineering. The energy evolution characteristics show that the proportion of dissipated energy is higher at low loading rates, and the proportion of elastic energy increases significantly at high loading rates. This law provides a new idea for disaster early warning in soft rock engineering: when the elastic energy inside surrounding rocks accumulates rapidly and the proportion of elastic energy (*U*_e_/*U*) increases continuously, it indicates an increased risk of unstable failure. Measures such as pressure relief and reinforcement should be taken in a timely manner to prevent disasters caused by instantaneous concentrated energy release.

This study reveals the effects of loading rate on the mechanical properties and energy evolution laws of rock-like specimens based on uniaxial compression tests, but fails to consider the confining pressure effect endured by deep rock masses in practice, which is inconsistent with the real triaxial stress environment. Confining pressure can significantly improve rock strength, alter failure modes, regulate energy distribution ratios, and weaken or change the strain rate response characteristics of materials. Therefore, the absence of confining pressure is the main limitation of this study. In the follow-up work, triaxial compression tests under the coupling effect of confining pressure and loading rate will be carried out to systematically reveal the effects of different confining pressure levels on the rate sensitivity, failure mechanism and energy evolution of rock-like materials. A mechanical response model more consistent with the stress environment of deep engineering will be established to provide more practical theoretical support for the stability control of deep soft rock roadways.

## 6. Conclusions

1) The density of rock-like specimens increases linearly with increasing cement-gypsum ratio, consistent with the density superposition principle for composite materials, and provides a quantitative reference for designing similar material mix ratios.
2) The stress-strain curves of rock-like specimens with different mix ratios all exhibit four-stage characteristics including compaction, elasticity, yielding and failure. The uniaxial compressive strength, elastic modulus and deformation modulus increase logarithmically with the increase of loading rate, and there is a significant coupling relationship between mechanical parameters and loading rate.
3) All specimens show columnar splitting failure. The stress concentration effect induced by force loading is more pronounced, leading to a greater degree of failure than that caused by displacement loading. A higher loading rate results in more sufficient crack development and poorer integrity of specimens after failure.
4) In terms of energy conversion, the lower the loading rate, the smaller the proportion of releasable elastic energy (*U*_e_/*U*) and the larger the proportion of dissipated energy (*U*_d_/*U*). Both parameters have a logarithmic function correlation with loading rate, and there is a significant coupling relationship between energy distribution pattern and failure mechanism.
